# Transactions of the Medical and Chirurgical Faculty of the State of Maryland

**Published:** 1891-03

**Authors:** 


TRANSACTIONS OF THE MEDICAL AND CHIRURGICAL FACULTY OF THE STATE OF MARYLAND, at its semi-annual session, held at Hagerstown, Md., November, 1889. Ninety-second annual session, held at Baltimore, Md., April, 1890.

The Transactions include papers on the following sections: Surgery; Practice of Medicine; Obstetrics and Gynecology; Materia Medica and Chemistry; Anatomy, Physiology, and Pathology; Psychology and Medical Jurisprudence; Ophthalmology, Otology and Laryngology.

The papers show great care in research. The subjects are well chosen, as the practical points of our science are those discussed. The paper on Review of Hypnotism, by Dr. Preston, is judicious and free from bias. The report of Punctured Fracture of the Cranium is a valuable addition in the literature of cranial surgery.

